# Leech Diversity in the Maghreb (North Africa): A Checklist and a Case Report of Parasitism on a Berber Toad (*Sclerophys mauritanica*) in Algeria

**DOI:** 10.3390/biology15090681

**Published:** 2026-04-26

**Authors:** Noureddine Rabah-Sidhoum, Mehdi Boucheikhchoukh, Bouthaina Hasnaoui, Mohammed Lamine Bendjeddou, Konstantinos Kostas, Noureddine Mechouk, Michail Kotsyfakis

**Affiliations:** 1 Institute of Molecular Biology and Biotechnology, Foundation for Research and Technology-Hellas, 70013 Heraklion, Crete, Greece; n.rabah-sidhoum@univ-eltarf.dz (N.R.-S.);; 2 Department of Veterinary Sciences, Chadli Bendjedid El Tarf University, PB 73, El-Tarf 36000, Algeria; 3 RITMES (Risques Infectieux Tropicaux Microorganismes Émergents), Aix-Marseille University, APhM, 13005 Marseille, France; 4 Institut Hospitalo-Universitaire Méditerranée Infection, 19-21 Boulevard Jean Moulin, 13005 Marseille, France; 5 Department of Parasitology and Parasitic Diseases, Faculty of Veterinary Medicine, University of Agricultural Sciences and Veterinary Medicine of Cluj-Napoca, Calea Mănăștur 3-5, 400372 Cluj-Napoca, Romania

**Keywords:** Hirudinea, leech, Maghreb, amphibians

## Abstract

Leeches (Hirudinea) are ecologically important annelids that interact with a wide range of aquatic vertebrates, yet their diversity, distribution, and epidemiological relevance remain poorly documented in North Africa. Here, we provide a comprehensive synthesis of freshwater and marine leech species reported from the Maghreb (Algeria, Tunisia, and Morocco), based on an extensive review of the available literature. In total, 21 species belonging to 13 genera and four families (Glossiphoniidae, Erpobdellidae, Hirudinidae, and Piscicolidae) are documented, with updated information on their ecology, host associations, and geographic distribution. In addition to this regional checklist, we report the first confirmed case of *Batracobdella algira* heavy parasitism on the Berber toad (*Sclerophrys mauritanica*) in Algeria. A single adult toad was found heavily infested by multiple leeches (*n* = 17), some of which bore spermatophores attached near the reproductive opercula, suggesting possible in situ mating behavior on the host. The high infestation observed in this single specimen may constitute an outlier, requiring further sampling to assess the effect of leeches on the anuran population in the region. By integrating faunistic data with a novel field observation, this study highlights the overlooked leech biodiversity in the Maghreb and suggests their possible ecological and epidemiological significance. Our findings emphasize the need for further investigations into leech–host interactions, pathogen carriage, and their implications for amphibian conservation and One Health in North Africa.

## Simple Summary

Freshwater ecosystems in North Africa remain poorly studied, and even conspicuous parasite groups, such as leeches, are overlooked despite their ecological roles and potential relevance to wildlife health. This study aimed to document leech biodiversity in the Maghreb with a case report of freshwater leech infestation. By reviewing the available literature, we compiled the first consolidated checklist of leech species from Algeria, Tunisia, and Morocco, identifying 21 species and highlighting major knowledge gaps in their distribution and host associations. We also report a new field observation of an unusually heavy leech infestation on a Berber toad in Algeria, representing a previously undocumented host–parasite interaction in the region. Although based on a single case, the high parasite load suggests that such interactions warrant greater attention and should be studied for their possible implications for amphibian health, especially in ecosystems under increasing environmental pressure. Our findings constitute an updated checklist of leech taxa in the Maghreb. By bringing attention to overlooked invertebrates and their interactions with vulnerable vertebrates, this study highlights the need for more integrative biodiversity surveys and long-term monitoring to better inform conservation strategies and freshwater ecosystem management in North Africa and in general.

## 1. Introduction

Leeches (Annelida: Hirudinea) comprise a diverse group of hematophagous and predatory annelids with a broad geographic distribution and the ability to exploit a wide range of aquatic and semi-aquatic habitats [1,2]. While predatory species represent a minor fraction of the group, the majority of leeches are blood-feeding ectoparasites that parasitize vertebrate hosts, including amphibians, reptiles, fishes, mammals, and occasionally humans [3,4]. Their parasitic lifestyle, coupled with prolonged host attachment and repeated feeding events, places leeches in a unique ecological position among invertebrate parasites.

Beyond their direct effects on hosts, hematophagous leeches have long been suspected of facilitating pathogen transmission. Several studies have reported their association with a wide diversity of microorganisms, including *Trypanosoma danilewsky* (Laveran & Mesnil, 1904) infecting carp and *Trypanosoma bubalisi* [5], as well as *Haemogregarina* spp., *Cryptobia* spp., *Bartonella* spp., *Rickettsia* spp., *Treponema* spp., *Clostridium* spp., *Aeromonas* spp., *Streptococcus* spp., and hog-cholera virus [6]. However, despite these reports, the epidemiological significance of leeches as vectors remains poorly understood, and host–parasite–pathogen interactions are rarely investigated in an integrated manner.

From an ecological perspective, amphibians are particularly relevant hosts, as they occupy key ecological niches, are highly sensitive to environmental change, and are increasingly threatened worldwide, especially by infectious agents. Although leech–amphibian associations have been documented in various regions, available data remain fragmented, geographically biased, and largely focused on Europe, parts of Asia, and northern America. In contrast, the leech fauna of North Africa, and the Maghreb in particular, remains insufficiently studied, with limited information on species diversity, host associations, and potential pathogen carriage [6,7,8,9,10].

In this context, the present study aims to synthesize existing data on leech taxa reported from the Maghreb and the bacterial, fungal, protozoan, and viral pathogens potentially associated with leeches, thereby addressing a regional knowledge gap. In addition, we provide original ecological observations, including the first documented record of *Batracobdella algira* (Moquin-Tandon, 1864) heavily infesting the Berber toad, *Sclerophrys mauritanica* (Schlegel, 1841), in Algeria.

## 2. Materials and Methods

A literature review of peer-reviewed publications has been conducted using the PubMed and Google Scholar databases. The extracted data up to 30 March 2025 included species name, host, geographical location, identification method, and associated microorganisms (the related data are represented in the Supplementary Material: Checklist of Maghrebin leeches). The included literature consisted of research articles reporting sampling effort, taxonomic observations, molecular biology assays such as DNA barcoding, and pathogen transmission experimental model studies. Gray literature materials, such as posters, conference papers, and theses, were systematically excluded. The keywords used were: Freshwater, Marine, Leech, Glossiphoniidae, Erpobdellidae, Hirudinidae, Piscicolidae, and Morocco, Algeria, or Tunisia. The observation site in Blida (north-central Algeria) was located at 36°26′45.0306″ N, 2°45′31.4958″ E. The recovered specimens were examined microscopically, following the morphological characteristics described by Ahmed and Tekaya [3]. The field observation reported herein was conducted independently of the literature survey and represents original empirical data.

## 3. Results

### 3.1. Regional Leech Diversity

#### 3.1.1. Freshwater Taxa, Ecology, and Distribution in the Maghreb

**Figure 1:**
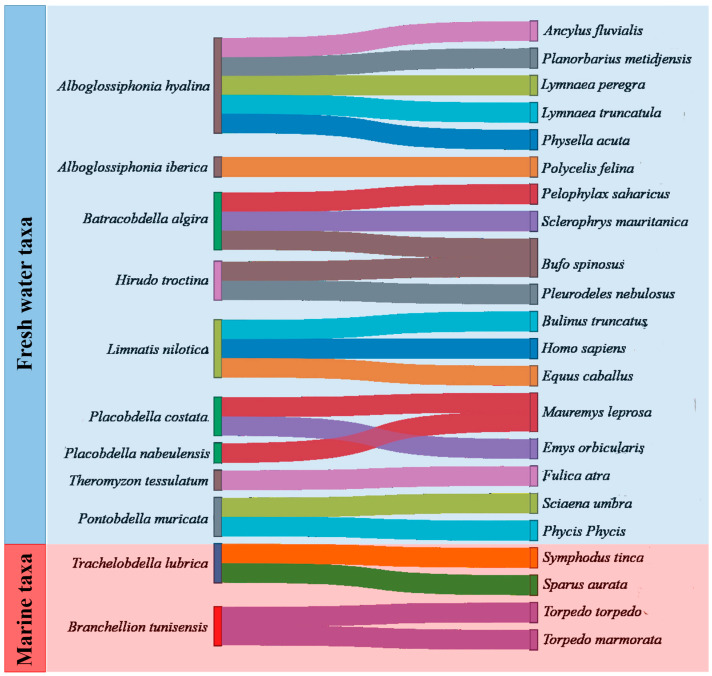
Associations between freshwater/marine leeches and their hosts, as recorded in previous studies across the Maghreb. The figure was generated from literature data compiled in the Supplementary Material (Checklist of Maghrebin leeches) using SankeyMATIC 7.3.0.

Twenty-one species from thirteen genera of leeches have been reported in the Maghreb (Figure 1). The majority of these species inhabit freshwater environments and are most frequently observed in free-living states, with only occasional associations with invertebrate or vertebrate hosts (Figure 1). Each species presents particular biological features and behavioral characteristics that are generally related to its host and environment.

##### Family Glossiphoniidae Vaillant, 1890

**Genus *Alboglossiphonia* Lukin, 1976**

The genus *Alboglossiphonia* is globally distributed, encompassing at least 25 species across all continents except Antarctica. The highest diversity is observed in Africa, where eleven species have been identified [7].

This genus encompasses small leech parasites of invertebrates, including mollusks and sponges. The species of this genus common in the Maghreb include *Alboglossiphonia hyalina* (O. F. Müller, 1774), which has been reported as one of the dominant species in soft, sandy-bottom, low-water-dynamics lakes [4,8,9].

This species, which prefers standing water and gentle current of artificial origin (e.g., from dams), can be found attached to the mantle of its hosts, such as *Ancylus fluviatilis* (Müller, 1774), *Planorbarius metidjensis* (Forbes, 1838), *Lymnaea peregra* (Müller, 1774), *Lymnaea truncatula* (Müller, 1774), and *Physella acuta* (Draparnaud, 1805), in the Moulouya River in Morocco as described by [10].

The presence of this species in Algeria cannot be ruled out, as it has been reported several times in neighboring countries. The second species is *Alboglossiphonia iberica* (Jueg, 2008), which occurs in stagnant waters poor in nutrients and oxygen-rich, such as springs and spring-fed streams. This leech is associated chiefly with isopods, followed by gastropods and planarians (*Polycelis felina* (Dalyell, 1814)).

**Geographic distribution**: although it has been recorded only in Morocco, its presence in Algeria and Tunisia is probable [3].

**Genus *Batracobdella* Viguier, 1879**

The genus *Batracobdella* is worldwide distributed, but represented solely by *B. algira* in North Africa. In Morocco, it was first described by Ahmed and Tekkaya [11] after examining the NMNH (National Museum of Natural History) collection in Paris. This species was first reported in Algeria and Tunisia one century ago under the name *Helobdella algira* (Seurat, 1922). However, its association with anurans was first documented by Ahmed et al. [4] based on samples collected in 1987 by Hotz in Béja, Tunisia, where it was observed parasitizing *Pelophylax saharicus* (Boulenger, 1913). *Sclerophrys mauritanica* (Schlegel, 1841) has also been reported as a host for this leech species in several regions of Tunisia: Nabeul [11] and east of Tabarka [12]. In Kabylia (Algeria), this species was found to infest *Bufo spinosus* (Daudin, 1803). As it happens, *B. algira* seems to parasitize anurans heavily, with a predilection for axillary and periocular sites [12].

This eurytopic freshwater leech can adapt to a wide range of habitats, including drainage basins, ooueds, springs, and marshes, and has been sampled from various regions in previous studies.

**Geographic distribution**: In Tunisia, it has been reported from Gafsa, Tozeur, Tebourba, El Haouaria, Nabeul, Beja, Djendouba, Bizerte, Kairouane, El Kef, Kroumiria, and Gbelli; in Morocco and Algeria, from the Moulouya River and Akfadou Forest, respectively [4,8,10,13,14,15,16].

**Genus *Helobdella* Blanchard, 1876**

The genus *Helobdella* comprises over 80 species that occur in several regions of the world, with the bulk of its diversity concentrated in South America, where more than 45 taxa have been identified [17,18,19].

This genus is represented by three Palearctic species in Maghreb (Table 1): *Helobdella stagnalis* (Linneaus, 1758), found in both Morocco and Tunisia; *Helobdella europaea* (Kutschera, 1987), described from Morocco; and *Helobdella octatestisaca* (Lai, 2009), reported recently from Tunisia and confirmed by molecular biology using COI-based DNA barcoding [10,20,21].

Although host associations have not been previously established in the Maghreb for this genus, some studies from other parts of the world have reported diverse interactions among *Helobdella* species. For instance, *Helobdella europea* parasitizes pond turtle *Emys orbicularis* (Linnaeus, 1758) in the Iberian Peninsula and Gulf Coast Box Turtle *Terrapene carolina major* (Linnaeus, 1758) in North America [22,23]. In 2017, Domínguez and Villarán [24] reported observations of an unidentified *Helobdella* sp. on *Mauremys leprosa* (Schweigger, 1812), which resembled *H. europea* but received no further investigation.

**Table 1:** Checklist of Hirudinea reported in Maghreb.

Leech Species	Authority Reference	Country	Identification	Source
*Alboglossiphonia hyaline*	O. F. Müller, 1774	M, T	Morph	[10]
*Alboglossiphonia iberica*	Jueg, 2008	M	Morph	[8]
*Batracobdella algira*	Moquin-Tandon, 1846	M, A, T	Morph	[11,15] Current study
*Branchellion torpedinis*	Savigny, 1822	A	Morph	[8]
*Branchellion tunisensis*	Youssef, Benmansour, Yurakhno & Mansour, 2024	T	Morph + COI and 18s rDNA	[25]
*Dina punctata maroccana*	Nesemann & Neubert, 1994	A, T	Morph	[26]
*Dina punctata punctata*	Johansson, 1927	M, A, T	Morph	[26]
*Erpobdella johanssoni*	Johansson, 1927	T	Morph + SEM	[27]
*Erpobdella testacea*	Savigny, 1820	T	Morph	[26]
*Helobdella europaea*	Kutschera, 1987	M	Morph	[10]
*Helobdella octatestisaca*	Lai, 2009	T	Morph + COI	[28]
*Helobdella stagnalis*	Linneaus, 1758	M, T	Morph	[8]
*Hirudo medicinalis*	Linnaeus, 1758	T	Morph + SEM	[27,29]
*Hirudo troctina*	Johnson, 1816	M, A, T	Morph + COI + SEM	[27,28,30]
*Limnatis nilotica*	Savigny, 1822	T	Morph + COI + SEM	[27,31,32]
*Placobdella costata*	F. Müller, 1846	M, A, T	Morph + COI and 12s rDNA	[8,33]
*Placobdella nabeulensis*	Ben Ahmed, 2023	A, T	Morph + COI and 12s rDNA	[4,34]
*Pontobdella muricata*	Linnaeus, 1758	T	Morph	[8]
*Theromyzon tessulatum*	O.F. Müller, 1774	T	Morph	[8]
*Trachelobdella lubrica*	Grube, 1840	A, T	Morph	[8]
*Trocheta Africana*	Nesemann and Neubert, 1994	T	Morph	[35]
*Trocheta tunisiana*	Ben Ahmed, 2013	T	Morph	[26]

M. Morocco, A. Algeria, T. Tunisia, SEM. Scanning Electronic Microscopy, Morph. Morphological Identification.

Regarding *H. stagnalis* ecology, it interacts with amphibians, usually found on the skin of its hosts, particularly *Bombina variegate* (Linnaeus, 1758), *Rana temporaria* (Rana temporaria), *Lissotriton helveticus* (Razoumowsky, 1789), *Ambystoma tigrinum* (Green, 1825), *Bufo bufo* (Linnaeus, 1758), *Ichthyosaura alpestris* (Laurenti, 1768), *Lissotriton vulgaris* (Linnaeus, 1758), and *Triturus cristatus* (Laurenti, 1768), to disperse in the environment. This qualifies its associations as phoretic rather than parasitic [36,37]. Additionally, *H. stagnalis* was described as a facultative mussel-associated species, with the interaction between the species unclear, described as simple inquilism or parasitism [38]. Similarly, the association of *H. stagnalis* with reptiles, namely the pond turtle *Emys trinacris* (Fritz, 2005), is not linked to nutritional reasons [39].

The third representative within this group (*H. octatestisaca*) was observed on *E. orbicularis* from the Iberian Peninsula [40]. In North America, this species was associated with different species of turtles (*Trachemys scripta* (Schoepf, 1792) and *Kinosternon subrubrum* (Bonnaterre, 1789)), frequently attaching to *Placobdella parasitica* (Say, 1824), suggesting that this relationship with turtles enhances their access to prey items [23]. This species was also reported on *Hyla eximia* (Baird, 1854) in southern America, raising questions on its possible facultative parasitism [41].

**Geographic distribution**: *H. stagnalis* was observed in the Moulouya, Selouane, and Melloulou rivers in Morocco [10] and in several streams in Tunisian governorates, namely El Kef, Siliana, and Bizerte [4,8,42]. As for *H. europea*, it was reported in the Sebou and Moulouya rivers, as well as in Selouane ditch lines, in Morocco [10,16].

**Genus *Placobdella* R. Blanchard, 1893**

The genus *Placobdella* is cosmopolitan and comprises 24 nominal species. However, this likely underestimates its true diversity due to a lack of informative morphological characters and the prevalence of inadequate or incomplete species descriptions [43]. Some species are currently being redescribed based on molecular biology studies [34].

The genus is represented by two species in the Maghreb. The first is *Placobdella costata* (F. Müller, 1846), a widely distributed species in the western Palearctic region, reported from the three countries. The second is *Placobdella nabeulensis* (Ben Ahmed, 2023), a new species found in extreme northeastern Algeria and Tunisia [34]. *P. costata* leeches were found several times on the Iberian pond turtle *M. leprosa* and *E. orbicularis*, as well as free questing within rivers, while *P. nabeulensis* only parasitized *M. leprosa* [4,8,33,34,44,45].

**Geographic distribution**: *P. costata* is distributed across the northern part of Tunisia, found in several lakes and rivers in the following governorates: Ghayadha, El Hania, Beni Toun, Jendouba, El Kef, and Nabeul [8,33,34]. As for Algeria, it is found in the Black Lake in Hchicha, Tonga Lake, Oued Bou Hchicha, and Brabtia in El Tarf [33,34,44]. In Morocco, this species is present in Zerouka Lake, Dar Bouazza, Oued Iriri, Oued Ksob, Oued Zat, Oued Tigrigra, Oued Sebou, Moulouya, and Melloulou Rivers [10,33,45].

Nonetheless, *P. nabeulensis* was reported only from the east of Algeria (Lake Tonga in El Tarf and Majen Belahriti Pond in Guelma) and Tunisia (Nabeul and El Malabi Dem in Menzel). However, the presence of this species in the rest of northern Algeria and Morocco is probable [8,34].

**Genus *Theromyzon* Philippi, 1867**

This genus occurs in the Holarctic realm, comprises 15 species, and has a broad global distribution, likely supported by its frequent presence in the nasopharyngeal cavities of migratory waterfowl, which facilitate long-distance dispersal [46,47,48]. There is only a single report of a *Theromyzon* species infesting *Fulica atra* (Linnaeus, 1758) in northeastern Algeria [49]. Additionally, in Nabeul, Tunisia, *Theromyzon tessulatum* (O.F. Müller, 1774) was sampled from the environment by Ben Ahmed [8]. This species resides in warm freshwater habitats, favoring stagnant waters [50].

##### Family Erpobdellidae R. Blanchard, 1894

**Genus *Dina* R. Blanchard, 1892**

The genus Dina is Holarctic; it consists of more than 25 taxa. In the Maghreb, it is represented by two conspecific subspecies: *Dina punctata punctata* (Johansson, 1927) and *Dina punctata maroccana* (Nesemann & Neubert, 1994). The first subspecies is known from southwestern Europe to the Maghreb. At the same time, the second is reported in several studies from the three considered countries since its first description in Ifran, Morocco [35]. Notwithstanding its phoretic association with vertebrates such as the spotted salamander *Ambystoma maculatum* (Shaw, 1802), host associations were not reported in the Maghreb or elsewhere, as this species exhibits a predatory behavior, feeding predominantly on Chironomidae larvae and Oligochaeta [51,52]. This species prefers eutrophic aquatic habitats, including creeks, small rivers, marshes, and temporary pools [8,10].

**Geographic distribution**: In Algeria, *D. p. punctata* was sampled from the Oued Dhimine in Oum El Bouaghi, while *D. p. maroccana* was found in the Arrafraf spring in the same province, Aïn Touta [4,8]. In Tunisia, *D. p. punctata* was found in Béja, Jendouba, Kairouan, Tozeur, Amdoun, Oued Er-Rmel in El Kef, Tfifila, Aïn Soltane, Aïn es Saniya, and Aïn Zakar in Siliana, Fernana, Bulla Regia, and Aïn Sidi Saleh in Bizerte. Meanwhile, *D. p. maroccana* originated from Aïn El Fejja in Bizerte and Aïn Nfaja, 24 km before Sejnane in Mateur [8].

**Genus *Trocheta* Dutrochet, 1817**

This genus is Palearctic, with several species occurring in Europe [26]. Siddall [53] formally synonymized it, along with the genera Dina and non-European *Mooreobdella* and *Nephelopsis*, under the genus *Erpobdella*. However, this classification has not been widely accepted, as many researchers have continued to recognize *Dina* and *Trocheta* as distinct genera [8].

Two freshwater predator species of this genus, *Trocheta africana* (Nesemann and Neubert, 1994) and *Trocheta tunisiana* (Raja Ben Ahmed, 2013) [26], which are associated with springs in elevated areas, are found in the Maghreb. Although these species have been recorded only in Tunisia, their presence in Algeria and Morocco is likely [8,26]. Indeed, Nesemann and Neubert [35] confirmed their presence in Algeria, noting that previous authors had misidentified them as *Dina quadristriata* (Grube, 1850) and *Dina lineata* (O. F. Müller, 1774).

**Geographic distribution**: In earlier studies, *T. a. africana* was found beneath river stones in Béja and Jendouba (northern Tunisia), as well as in a stream near Hammam Bourguiba in Kroumirie and the El Feïja River [8,26,35]. As for *T. tunisiana*, it has been reported from several localities, including Oued El Madina, Oued El Melah, Ouechtata, Aïn Es-Sobh spring in Jendouba, a spring in Aïn Draham, as well as locations in Béja and Jendouba [8,26,28,53,54,55,56,57].

##### Family Hirudinidae Whitman, 1886

**Genus *Hirudo* Linnaeus, 1758**

*Hirudo*, the genus of medicinal leeches, is found worldwide and comprises about six species [58]. This group has two recognized representatives in the Maghreb. *Hirudo troctina* (Johnson, 1816) and *Hirudo medicinalis* (Linnaeus, 1758). The first was recorded in the three countries, whereas the second was reported only in Tunisia. However, the presence of *H. medicinalis* in Algeria and Morocco may be admitted, given that it is a cosmopolitan species [59]. Several clinical cases from Tunisia and Morocco reported the presence of leeches in the upper aerodigestive tracts of hospitalized patients, but none provided precise identification. Some studies included photographs of what can be considered *Hirudo* species [29,60,61]. Nevertheless, the precise description of this species was provided by Boughalmi et al. [31] after sampling from the Oued Sarrath in Tunisia. Moreover, advanced techniques, including scanning electron microscopy, were used to characterize samples of this species from Tunisia [27].

Regarding the dragon leech *H. troctina* parasitism, observations on a spined toad *B. spinosus* and a dead Algerian ribbed newt *Pleurodeles nebulosus* (Guichenot, 1850) were reported from Akfadou forest in Tizi Ouzou, Algeria, which suggests that this species admits various hosts and can be influential in the regression of endangered species populations [15,62].

**Geographic distribution**: In Tunisia, *H. troctina* was found at Lebna Dam and Mlaabi Dam, as well as in streams from Tabarka, Aïn Draham, Nabeul, Jendouba, Kroumiria, Sousse, and Tozeur [3,8,13,21,27,30]. This species was recorded in different locations in Morocco, including the Moulouya and Melloulou rivers, Aguelman Lake in Sidi Ali, the Oum Errabiâ River, and the south-west of Azrou [10,30].

**Genus *Limnatis* Moquin-Tandon, 1826**

This group is Palearctic and contains three species: *Limnatis paluda* (Tennent, 1859), known from the Asian continent; *Limnatis nilotica* (Savigny, 1822), well known in Africa; and *Limnatis bacescui* (Manoleli, 1972), endemic to southern Europe [32,63,64]. Only one species of this genus is encountered in the Maghreb, *L. nilotica*, the Nile leech, which has been reported from Morocco to Libya [8,10,32,65]. Thus, it is present throughout western North Africa. Adults of this hematophagous species need a blood meal during the mating season to breed and lay their cocoons [65]. Individuals of this species are very generalists and feed on a wide range of taxa. They are considered voracious predators of invertebrates, including *Bulinus truncatus* (Audouin, 1827) snails [66], and parasites of vertebrates, including amphibians, animals, and humans [67,68]. They are important from both ecological and epidemiological perspectives.

**Geographic distribution**: *L. nilotica* has been recently reported in Ain Tafrent spring, Debdou and Beni Bouayach mountains, Sidi Moussa, Jbel Mahser and Beni Ouaklane, Gafait spring, and downstream in the Moulouya River at Sabra and Zegzel springs, Azougagh and Driouch in Morocco [10,32]. In Tunisia, it was collected from streams in Bizerte, Ariana, Ben Arous, Béja, Siliana, Zaghouan, Nabeul, Gabes, Jendouba, Kairouan, Gafsa, and Tozeur governorates [4]. As for Algeria, the species was recovered from a man who presented to Saïda hospital [69].

#### 3.1.2. Marine Taxa, Ecology, and Distribution in the Maghreb

In contrast to freshwater taxa, the description of marine leeches from the Maghreb is scarce. In fact, only four species belonging to three genera have been described from this region.

##### Family Piscicolidae Johnston, 1865

**Genus *Pontdobdella* Leach, 1815**

This genus is globally distributed and encompasses 14 marine species, according to recent data [70]. One of the few reported species is *Pontobdella muricata* (Linnaeus, 1758)—a parasite of several species in the Mediterranean region in general, including elasmobranchs (*Raja miraletus* (Linnaeus, 1758), *Raja montagui* (Fowler, 1910), *Raja clavata* (Linnaeus, 1758), *Torpedo marmorata* (Risso, 1810), *Dipturus oxyrinchus* (Linnaeus, 1758), and *Dasyatis pastinaca* (Linnaeus, 1758))—and other fishes, including *Sciaena umbra* (Linnaeus, 1758) and *Phycis phycis* (Linnaeus, 1766), whose infestation episodes were observed from Tabarka coast in Tunisia, with a predilection to gills, fins, and skin [71,72,73,74,75,76]. This species was also reported in the free state from Oran, Algeria, and Morocco [8].

**Geographic distribution**: Mediterranean Maghrebin coast.

**Genus *Trachelobdella* Deising, 1850**

This group has undergone several reevaluations of its taxa; it now comprises some nine species of marine leeches, with one taxon inquirendum (i.e., *Trachelobdella alcibiades* (Leigh-Sharpe, 1933)). This genus is cosmopolitan [77,78,79,80,81]. The common leech species of the Maghreb coast is *Trachelobdella lubrica* (Grube, 1840), which has a worldwide distribution. The infestation by this marine taxon has been recorded on several Mediterranean fish species, including *Dicentrarchus labrax* (Linnaeus, 1758), *Serranus cabrilla* (Linnaeus, 1758), *S. umbra*, and *Scorpaena porcus* (Linnaeus, 1758) [82,83,84,85]. In Tunisia, free samples were found in Bizerte Lagoon and parasitizing *Symphodus tinca* (Linnaeus, 1758) [4,8]. Whereas in the Béjaïa coast (eastern Algeria), this species was collected from the gills of *Sparus aurata* (Linnaeus, 1758) [86].

**Geographic distribution**: Mediterranean Maghrebin coast.

**Genus *Branchellion* Savigny, 1822**

*Branchellion* is a genus of marine leeches comprising around a dozen species that are strictly hematophagous and parasitic on chondrichthyans, primarily batoid fishes worldwide. They are typically found on the body surface and within the orifices of their hosts [70]. One species (*Branchellion torpedinis* (Savigny, 1822)) from Algeria is deposited at the MNHN of Paris by M. Hollard [8]; this species parasitizes mainly elasmobranchs [87]. The second species is *Branchellion tunisensis* [25], a new species, recently discovered through morphological identification and DNA barcoding using COI and 18S markers. This new species was identified in a study of *Torpedo torpedo* (Linnaeus, 1758) and *Torpedo marmorata* (Risso, 1810) in the Tunisian Gulf [25].

**Geographic distribution**: Mediterranean Maghrebin coast.

### 3.2. Field Evidence of Intense Batracobdella algira Parasitism in Algeria

On the late evening of 24 May 2023, an adult female *S. mauritanica* was found exhibiting signs of lethargy and was closely examined, revealing numerous leeches attached to both axillae, with a few also attached to the eye area and the ventral region of the head. Leeches (n = 17) were subsequently collected. Then, preserved in 70% ethanol and transported to the Biodiversity and Ecosystems Pollution Laboratory at Chadli Bendjedid University for morphological identification based on specific descriptions provided by Ahmed [11].

**Figure 2:**
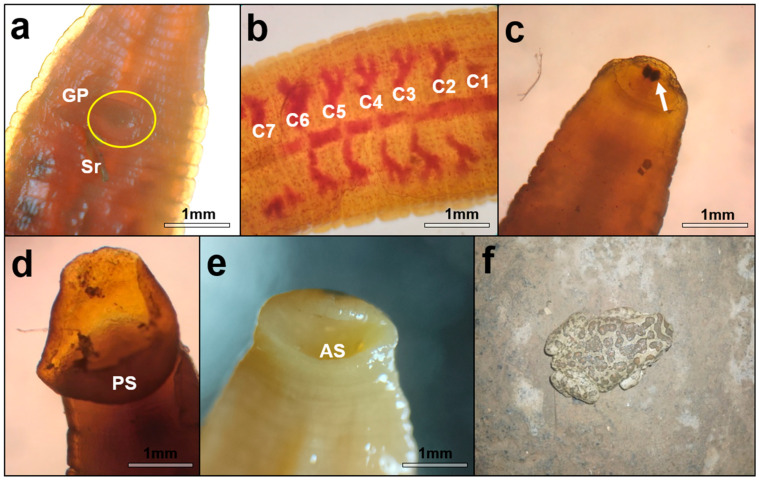
Morphological characteristics of the recovered specimens of *B. algira*. (**a**) Spermatophore (Sr) tightly attached to the ventral side of the leech, adjacent to the gonopores (GP) in circle. (**b**) Dorsal view of the seven pairs of caecal crops (C1–C7) filled with anuran blood. (**c**) Cephalic region showing separate, simple eyes. (**d**) Close-up view (×40) of the posterior sucker (PS). (**e**) Close-up view (×40) of the proboscis (AS). (**f**) Photograph of the infested Berber toad following sampling.

Photographs of the parasitized toad, along with microscopic observations of one of the parasites, are presented in Figure 2. The individuals varied in size; however, accurate morphometric measurements were not possible due to specimen shrinkage caused by direct fixation without prior relaxation. Microscopic examination revealed that some individuals had one or more spermatophores attached to their ventral side, particularly near the reproductive opercula. However, it remains unclear whether this reproductive behavior occurred on the sampled host or before sticking to it.

Besides similar observations by Ben Ahmed [11] and Beukema [12] in Tunisia, predation by *S. mauritanica* on *B. algira* has not been reported in the Maghreb region, in general or specifically in Algeria. However, the predation of *B. spinosus* by *B. algira* was documented by Merabet et al. [15] in the Akfadou Forest, Bejaia. Notably, our observation of *S. mauritanica* parasitized by *B. algira*, with an unusually high number of leeches found on a single individual, is the first of its kind in Algeria.

## 4. Discussion

Although several species of Hirudinea are hematophagous parasites, the diseases they may transmit remain poorly studied. Previous research on species in the Maghreb has mainly focused on experimental models, particularly medicinal leeches of the genus *Hirudo*, as potential carriers of nosocomial infection agents. Apart from the study on *P. costata* associated with *M. leprosa* and *E. orbicularis*, which revealed the presence of *Haemogregarina stepanowi* in the Maghreb [33], no other surveys have investigated the pathogen load of leeches in this region.

However, studies on *H. verbana*, *H. nipponia*, and *H. manillensis* have enabled the isolation of several bacterial species, including *Chryseobacterium gleum*, *Ochrobactrum anthropi*, *Moraxella osloensis*, *Microbacterium oxydans*, *Kytococcus sedentarius*, *Rhizobium radiobacter*, *Staphylococcus hominis*, *Klebsiella pneumoniae*, *Aeromonas veronii*, *Citrobacter* spp., and *Bacillus* spp. In addition, multiple fungal species have been identified, such as *Candida albicans*, *Candida ciferrii*, *Candida famata*, *Candida guilliermondii*, *Candida parapsilosis*, *Lipomyces starkeyi*, *Rhodosporidium* sp., *Schizosaccharomyces* sp., *Trichosporonoides oedocephalis*, and *Yarrowia lipolytica* [88,89,90]. Given these findings, the presence of similar microorganisms in Maghrebin *Hirudo* spp. populations represents a plausible hypothesis.

Candida agents have also been identified in *Theromyzon T. maculosum*, an avian blood-feeding leech closely related to *T. tessulatum*, including *C. albicans*, *C. glabrata*, and *C. krusei* [91]. Some viruses can persist in leeches and be mechanically transmitted per os, such as African swine fever virus (ASFV) [92]. Furthermore, serological evidence of human immunodeficiency virus (HIV) and hepatitis B virus (HBV) has been reported in *H. medicinalis* specimens from Cameroon [6]. The presence of viral iDNA in leeches and eDNA in their environment has also revealed a wide range of mammalian-related viruses, including Rhabdoviridae, Coronoviridae, Anelloviridae, Parvoviridae, and Circoviridae (Alfano [93], highlighting their potential role in viral persistence.

Kinetoplastids are also commonly associated with leeches. While some species, such as *Trypanoplasma borreli*, survive only a few days in the leech crop (up to 14 days before digestion), other mammalian-related species, including *Toxoplasma gondii*, *Trypanosoma brucei brucei*, and *Plasmodium berghei*, can reproduce as long as ingested blood cells are preserved [6,94]. Ray [95] described the development of *Trypanosoma balithaensis* in *Helobdella nociva*, while *Trypanosoma cobitis* was later shown to reproduce experimentally in *Hemiclepis marginata* (Lewis and Ball, 1980). In colder Palearctic regions, a *Trypanosoma* species was found to reproduce in and be transmitted by *Johanssonia arctica* to various fish species [96]. More recently, Smit et al. [97] reported the molecular characterization and development of *Trypanosoma mukasai* in *Batracobdelloides tricarinata*. Su et al. [98] described a new mammalian parasite species, *Trypanosoma (Megatrypanum) bubalisi*, in *Hirudinaria manillensis*, which feeds on wild water buffalo (*Bubalus bubalis*).

Research on other leech species, particularly from the Far Eastern Palearctic, has also demonstrated their capacity to harbor bacterial pathogens such as *Rickettsia* spp., *Pseudomonas fluorescens*, *Enterobacter agglomerans*, *Flavobacterium indologens*, *Aeromonas hydrophila caviae*, *Vibrio hollisae*, and *Bartonella grahamii* [99,100,101]. Although our data do not confirm active pathogen transmission in the Maghreb, they highlight plausible epidemiological scenarios and the need for experimental validation to better assess leech vector competence and epidemiological significance.

Over the last few decades, the Maghreb region has become a hotspot for climate change impacts [102]. Global warming, deforestation, and the region’s geographic position have contributed to a marked decrease in cold-season precipitation in Algeria and Morocco [103]. At the same time, rapid population growth, mainly concentrated in northern areas, combined with economic development, has increased water demand and intensified water stress [104]. Consequently, local populations increasingly rely on non-conventional water sources for drinking and irrigation, increasing interactions between humans and freshwater ecosystems, including leech habitats.

Human activities strongly affect leech habitats and reproductive environments. Habitat fragmentation, evaporation of lakes and temporary water bodies, and agricultural pollution can significantly alter freshwater ecosystems [105]. These pressures may reduce leech dispersal, leading to smaller, more unstable populations. Simultaneously, human disturbance negatively affects local anuran abundance and disrupts their movement and dispersal [106]. This shift in host availability may result in increased infestation intensity, as observed in the present study, where a single toad was heavily infested by 17 leech individuals in a disturbed oued surrounded by buildings and agricultural activities.

The ecological role of leeches in regulating anuran populations can be considered a double-edged sword. Under natural conditions, leeches may contribute to population control and ecosystem balance [14]. However, in fragmented habitats with limited water surfaces, high infestation pressure may exacerbate instability in already vulnerable anuran populations [107]. In such contexts, parasitism may act as an additional stressor rather than a regulatory mechanism.

This single observation provides a preliminary record of leech infestation in the understudied Chiffa region. However, its broader ecological significance cannot be determined without additional data. Thus, a greater sampling effort should be directed toward the fragmented Chiffa stream as part of long-term monitoring to assess amphibian conservation status and their interactions with Glossiphonidae.

## 5. Conclusions

This study combines a regional synthesis of Hirudinea diversity in the Maghreb with the first recorded observation of *Batracobdella algira* parasitizing the Berber toad (*Sclerophrys mauritanica*) in Algeria, shedding light on an overlooked host–parasite interaction. Additionally, the unique toad examined was heavily parasitized, with an unusually high number of leeches present. Although based on a single host individual, the exceptionally high infestation intensity and associated reproductive indicators provide biologically meaningful evidence of a previously undocumented interaction.

We also compiled a list of 21 leech species in Algeria, Tunisia, and Morocco based on literature records, contributing to a broader understanding of leech diversity and distribution in North Africa.

From an epidemiologic perspective, the implications of leech parasitism can be summarized in two key points: (1) The transmission of pathogens by freshwater leeches is well documented compared to that by marine leeches. (2) Most pathogens of zoonotic importance are detected in generalist blood feeder leeches of the genus *Hirudo*, but experimentation is required to resolve any susceptible role in their transmission.

While the direct impact of leech parasitism on amphibian populations remains unclear, these findings highlight the need for further research into their ecological and epidemiological significance.

## Data Availability

The original contributions presented in this study are included in the article/Supplementary Material. Further inquiries can be directed to the corresponding author.

## Supplementary Materials

The following supporting information can be downloaded at: https://www.mdpi.com/article/10.3390/biology15090681/s1. References [108,109,110,111,112,113,114,115,116,117,118,119,120,121,122,123,124] are cited in the Supplementary Materials.
